# Genetic loci associated with an earlier age at onset in multiplex schizophrenia

**DOI:** 10.1038/s41598-017-06795-8

**Published:** 2017-07-25

**Authors:** Annemarie L. Woolston, Po-Chang Hsiao, Po-Hsiu Kuo, Shi-Heng Wang, Yin-Ju Lien, Chih-Min Liu, Hai-Gwo Hwu, Tzu-Pin Lu, Eric Y. Chuang, Li-Ching Chang, Chien-Hsiun Chen, Jer-Yuarn Wu, Ming T. Tsuang, Wei J. Chen

**Affiliations:** 1 National Taiwan University, College of Public Health, Institute of Epidemiology and Preventive Medicine, Taipei, 100 Taiwan; 2 National Taiwan University, Genetic Epidemiology Core, Center of Genomic Medicine, Taipei, 100 Taiwan; 3 National Taiwan University, College of Public Health, Research Center of Genes, Environment, and Human Health, Taipei, 100 Taiwan; 4 China Medical University, Graduate Institute of Biostatistics, Taichung, 404 Taiwan; 5 National Taiwan Normal University, College of Education, College of Education, Department of Health Promotion and Health Education, Taipei, 106 Taiwan; 6 Department of Psychiatry, College of Medicine and National Taiwan University Hospital, National Taiwan University, Taipei, 100 Taiwan; 7 National Taiwan University Hospital, College of Medicine, Graduate Institute of Brain and Mind Sciences, National Taiwan University, Taipei, 100 Taiwan; 8 National Taiwan University, Center of Genomic Medicine, Bioinformatics and Biostatistics Core, Taipei, 100 Taiwan; 9 National Taiwan University, College of Electrical Engineering and Computer Science, Graduate Institute of Biomedical Electronics and Bioinformatics, Taipei, 106 Taiwan; 10 Academia Sinica, Institute of Biomedical Sciences, Taipei, 115 Taiwan; 11 University of California, Center for Behavioral Genomics and Institute for Genomic Medicine, Department of Psychiatry, San Diego, 92093-0761 CA USA; 12 University of California, Department of Psychiatry, San Diego, 92093-0603 CA USA; 13 Harvard Institute of Psychiatric Epidemiology and Genetics, Boston, 02115 MA USA

## Abstract

An earlier age at onset (AAO) has been associated with greater genetic loadings in schizophrenia. This study aimed to identify modifier loci associated with an earlier AAO of schizophrenia. A genome-wide association analysis (GWAS) was conducted in 94 schizophrenia probands with the earliest AAO and 91 with the latest AAO. Candidate single nucleotide polymorphisms (SNPs) were then genotyped in the co-affected siblings and unrelated probands. Multi-SNP genetic risk scores (GRS) composed of the candidate loci were used to distinguish patients with an early or late AAO. The 14-SNP GRS could distinguish the co-affected siblings (n = 90) of the earliest probands from those (n = 91) of the latest probands. When 132 patients with an earlier AAO and 158 patients with a later AAO were included, a significant trend in the 14-SNP GRS was detected among those unrelated probands from 4 family groups with the earliest, earlier, later, and latest AAO. The overall effect of the 14 SNPs on an AAO in schizophrenia was verified using co-affected siblings of the GWAS probands and trend effect across unrelated patients. Preliminary network analysis of these loci revealed the involvement of *PARK2*, a gene intensively reported in Parkinson’s disease and schizophrenia research.

## Introduction

Schizophrenia has long been described as a complex disorder and its aetiology remains elusive^1–3^. Family and twin studies have indicated that genetic factors contributed substantially to schizophrenia, with an estimated heritability of approximately 80%^4^. When hundreds of microsatellite markers were examined in genome-wide linkage studies (GWLS) to look for genetic variants that co-segregated with the illness within families, suggestive evidence for a dozen of linkage regions was found^5^. With the advent of genome-wide association studies (GWAS), in which hundreds of thousands of single nucleotide polymorphisms (SNPs) were examined, approximately 100 markers conferring small increments in risk have been found in schizophrenia^6, 7^.

Nevertheless, the combined variance explained by the SNPs reaching genome-wide significance remained low^6^. Alternatively, an application of GWAS data, the polygenic risk score (PRS), has been discovered^6^. The PRS, consisting of SNPs with P-values less than certain thresholds in GWAS, has been demonstrated to provide clues to functional analysis^6, 8^. Examination of the PRS derived from the results of large-scale GWAS^6, 7^ has been used to predict individuals with first-episode psychosis from controls^9^ and those predisposed to schizophrenia in adolescence^10^. The associations between the PRS and some clinical dimensions of schizophrenia^11^ and first-episode psychosis^12^ have also been reported.

In search of the genetic factors contributing to schizophrenia, another class of genes involved in the clinical manifestations of the illness, i.e., modifier genes, also needs to be considered^13^. Modifier loci associated with clinical features, such as the age at onset (AAO)^14, 15^ and positive and negative symptoms^16^ but not with schizophrenia susceptibility per se, have been identified using GWAS. Furthermore, our previous ordered subset linkage analysis identified a prominent linkage peak in a subset of schizophrenia patients characterized by earlier AAO^17^.

In this study, we aimed to identify genetic loci associated with earlier AAO of schizophrenia in multiplex families from a single ethnicity. We performed a case-only GWAS for AAO of schizophrenia in a multiplex family sample of Han Chinese in Taiwan. Individual SNPs above certain significance thresholds were further tested in two subsamples, including co-affected siblings and other unrelated patients from the same recruitment scheme. Then, a multi-SNP genetic risk score (GRS) was applied to estimate an overall effect of the candidate genetic loci. The influence of the AAO-modifying genetic loci on the susceptibility of schizophrenia was also evaluated to clarify the potential effect using additional population-based case-control and family-based association tests. Finally, candidate SNPs were subjected to gene network analysis to explore their possible functions associated with the AAO of schizophrenia.

## Materials and Methods

### Participants

The multiplex family samples used in this study were selected from the Taiwan Schizophrenia Linkage Study (TSLS). First-degree relatives from Han Chinese families with more than two children affected by schizophrenia were recruited across Taiwan. Detailed methods of the fieldwork have been described elsewhere^18^. Briefly, interviews at recruitment were performed using the Diagnostic Interview for Genetic Studies (DIGS)^19^, accompanied with the Family Diagnostic Interview for Genetic Studies (FIGS)^20^. Research assistants carried out a Chinese version of the DIGS, following standard training as described by Chen and colleagues^21^. The diagnosis of schizophrenia was made independently by at least two research psychiatrists based on the criteria of the fourth edition of the Diagnostic and Statistical Manual (DSM-IV), joined with the record of DIGS, FIGS, interviewer notes, and hospital anamnesis. Information regarding an AAO of the first psychotic episode was extracted from the psychosis section of DIGS, or from the medical history where necessary. Whole blood samples were collected and sent to the National Institute of Mental Health (NIMH) Repository and Genomics Resource (RGR) to be transformed into lymphoblastoid cell lines and stored. DNA samples were extracted from the cell lines and then transported to Taiwan.

The study design and selection of family samples are illustrated in Supplementary Figure S1. Among the 607 families recruited by the TSLS^18^, 2242 individuals from 557 multiplex families, including 1207 affected (1150 siblings, 57 parents) and 1035 unaffected (271 siblings and 764 parents) individuals, were successfully genotyped in a genome-wide linkage analysis^22^. Previous GWLS of these multiplex families indicated that a subset of families with early AAO led to a significant linkage signal^17^. Hence, we postulated that a GWAS screening based on a subsample with extreme contrast in AAO might maximize the power to identify candidate modifier genetic variants. Nevertheless, the chosen variants could then be genotyped in the remaining samples for the confirmation of the association with AAO. First, the 557 families were ranked according to the average AAO of the siblings affected by schizophrenia from each family. Second, a power evaluation of a two-group design (patients with extremely early AAO vs. patients with extremely late AAO) with 80 patients in each group reached 0.7–0.9 using Genetic Power Calculator^23^ under the following conditions: the minor allele frequencies of the SNPs are 0.2–0.45; the genotype relative risk for heterozygote and homozygote are 1.9–2.0 and 2.0–2.2, respectively; and the prevalence of early AAO among patients on average is 0.3–0.5, according to our empirical data. The final sample size increased from 80 to 95 per group since the microarray chips used in this study could hold 190 samples. Hence, the 95 families with the earliest average AAO and the 95 families with the latest average AAO were identified as the subsample with an extreme contrast in the AAO. One proband with an AAO conforming to the contrast from each family (i.e., earlier AAO in the extremely early subgroup and later AAO in the extremely late subgroup) was selected for GWAS genotyping. After sample quality control (discussed later), 185 probands (94 of the earliest AAO subgroup and 91 of the latest AAO subgroup) remained. SNPs with the strongest association signals (P-value < 10^−4^) in the association tests were identified as a set of candidate SNPs. Third, the co-affected siblings (n = 181), whose DNA material were available, from the initial 185 families were then genotyped for the consideration that their genetic background was similar to the initial probands.

Another two subgroups of schizophrenia patients were selected from the remaining families with less contrast in average AAO. However, the number of patients that could be genotyped was determined by the slots available for candidate loci genotyping after the co-affected sibling and parents of the initial 185 probands, i.e., at most 300 samples. Using similar principles in selecting one patient from each family as before, 132 patients from families of earlier AAO and 158 patients from families of later AAO were then genotyped for the candidate SNPs. Along with the 185 initial probands with extremely early AAO (n = 94) and extremely late AAO (n = 91), those unrelated schizophrenia patients could be divided into four subgroups (the earliest, earlier, later, and latest AAO) and exhibited an increasing trend of average AAO (Supplementary Figure S2). This four-group contrast provided an opportunity to examine a gradient effect of the candidate loci on AAO. The remaining 77 families with modest contrast in the average AAO were not included for the genotyping of candidate loci in consideration of the efficiency of the trend analyses.

To evaluate whether the candidate SNPs associated with AAO were also involved in susceptibility for schizophrenia, two strategies of association analysis were adopted. The first strategy was to genotype the parents (n = 288) from the initial 185 probands and then to pool them with the affected siblings to undergo family-based association test for schizophrenia. The second strategy was to conduct a case-control association analysis. Community controls free of major mental disorders were selected from the Han Chinese Cell and Genome Bank in Taiwan (HCCGB)^24^ using a frequency matching in age and gender distribution with a 5 to 1 ratio of controls versus cases^25^. A total of 925 community controls were selected to match the 185 probands with genome-wide SNP data (Supplementary Figure S1).

### SNP genotyping and quality controls

The genome-wide SNP genotyping was performed on 190 schizophrenia probands of extreme contrast in AAO using the Axiom Genome-wide CHB 1 Array Plate (Affymetrix, Santa Clara, CA, USA). A total of 642,832 SNP genotypes were examined. Quality control of the samples and markers was performed according to the standard procedure for GWAS^26^, and was mainly performed using PLINK 1.07^27^. Briefly, SNP data from each proband were checked for gender inconsistency and sample genotyping call rate (>98%).

All genetic markers were checked for marker genotyping call rate (>98%), minor allele frequency (>0.05), and Hardy-Weinberg equilibrium (HWE) with a P-value of >0.001. For population stratification, or check for the homogeneity of the sample, we followed the procedures provided by PLINK. The whole-genome identical by state (IBS) distances were firstly calculated between all individuals. Four multidimensional scaling (MDS) components of each individual were then estimated using the Hamming distance. A MDS component with a significant difference in the comparison would be adjusted as a covariate in the GWAS analyses. Relatedness between probands was estimated based on pairwise kinship using the KING software^28^. Batch effects were also checked and adjusted. Finally, 185 probands with schizophrenia remained after the quality control measures, with 5 patients being deleted due to low call rates (n = 2), a high kinship with another proband (n = 1), or population stratification suggesting different ancestry (n = 2). A total of 564,836 SNPs remained after the quality control. The overall genotyping error was 0.42%. Afterwards, the genomic inflation factor was very small (λ = 1.0013).

Genotyping for the candidate SNPs in available co-affected siblings and parents and an independent set of schizophrenia probands with less contrast in AAO was carried out using the Illumina GoldenGate Genotyping Assay, which allows for customising probes of the SNPs of interest. The SNP genotyping of the community controls was performed by the biobank using the same microarray chip (Affymetrix Axiom Genome-wide CHB 1 Array Plate) as used for the schizophrenia probands. Only the data of the candidate SNPs in the community controls are presented in this study.

### Data analyses

A two-tailed Fisher’s exact test for dichotomous variables and a two-tailed t-test for continuous variables were used for group comparisons. Multiple logistic regression with adjustment for one of the four MDS components was used for association analysis for individual SNPs between GWAS probands from families with extreme contrast in AAO. We did not include covariates such as age and gender in the regression models to increase the power when searching for new genetic loci in diseases with low prevalence like schizophrenia^29^. Because age and AAO of the patients in the TSLS sample (n = 1,285) were highly correlated, with a Pearson’s correlation coefficient (r) = 0.52, we did not adjust for age in subsequent association analyses in the data sets of individual genotyping. Furthermore, we did not adjust for gender in these analyses since only autosomal SNPs were included in our GWAS analysis. Multiple comparisons of the association tests on the candidate SNPs were adjusted using Bonferroni correction. A P-value lower than 7.2 × 10^−8^ was considered the threshold of genome-wide significance^30^, while a less stringent threshold of P-value < 10^−4^ was used to filter SNPs with the strongest association. Owing to these considerations, simple logistic regression analyses were used for the analysis of the co-affected siblings of the initial GWAS probands. Ordinal logistic regression was used to examine whether there was a linear trend in the allele frequency of a candidate SNP across schizophrenia probands from the earliest, earlier, later, and latest AAO family subgroups. Nevertheless, to exclude potential confounding by gender we also performed alternative statistic models with adjustment for gender in the analysis of co-affected siblings and the 4 groups of unrelated probands.

Multi-SNP GRS was constructed by sum of logarithm of odds ratio (logOR) multiplied by genotypes (coded as 0, 1 and 2) of the candidate SNPs with 0 for the homozygosity of major allele, 1 for the heterozygosity, and 2 for homozygosity of minor allele. The GRS were estimated using the logOR from the GWAS analysis of 185 probands and presented as the means ± standard error. Comparisons between the earliest and the latest AAO family subgroups using non-parametric Mann-Whitney U-test were performed separately among the probands and their co-affected siblings. The trend test for the GRS between the four AAO family subgroups was carried out using linear regression.

The influence of the AAO-associated SNPs on the susceptibility to schizophrenia was evaluated using both family-based and population-based designs. First, for those families with SNP genotyping information, family-based association analyses were performed using software family-based association test (FBAT) version 2.0.3^31^, with affected status as outcome and the additive model for genotypes. Second, the schizophrenia probands with GWAS genotyping and their age- and gender-matched community controls were subjected to association analysis using multiple logistic regression with adjustment for age and gender to avoid selection bias.

The possible function of a SNP was predicted by the GoldenPath as described in the F-SNP database (http://compbio.cs.queensu.ca/F-SNP). Then, those candidate SNPs with gene annotation were subjected to network analyses using the Ingenuity Pathway Analysis (IPA; http://www.qiagen.com/ingenuity). The significance level of the gene network analysis was determined using -log10(P-value) and noted as the p-score.

### Ethics statement

This study has been approved by the National Taiwan University Hospital Research Ethics Committee (NTUH-REC, No.: 201003106 R and 201411086RINC) for the analyses including all relevant details. The use of personal information and DNA samples for genotyping experiments were performed in accordance with the NTUH-REC guidelines and regulations. The sample collections of families with schizophrenia and normal controls were approved by the National Taiwan University Hospital’s Internal Review Board of Human Studies and the Internal Review Board of the Institute of Biomedical Sciences, Academia Sinica as described in the articles of the filed work^18, 24^.

### Data Availability

The datasets generated during and/or analysed during the current study are available from the corresponding author on reasonable request.

## Results

For the 185 families with extreme contrast in AAO, the difference in AAO for both the probands and their co-affected sibling subsamples were significant between families with the earliest AAO and those with the latest AAO (Table 1). Further, the difference in AAO for an independent set of probands (n = 290) from other families with less contrast in AAO was also significant. For those families with affected parents, the average AAO of the affected parents from families with the earliest AAO was younger than those with the latest AAO, and parental AAOs were later than their affected children, regardless of family subgroups. The distribution of gender did not differ among groups. However, the average age of the members from families with an early AAO was statistically younger than that of the families with an early AAO.

**Table 1:** Comparison of gender, age, and age at onset distribution of the family samples with extreme and less contrast in the age at onset.

		Extreme contrast in the AAO							Less contrast in the AAO						
		All		Earliest AAO		Latest AAO		P-value	All		Earlier AAO		Later AAO		P-value
Probands		n = 185		n = 94		n = 91			n = 290		n = 132		n = 158		
	Male, n (%)	117	(63%)	53	(56%)	64	(70%)	0.06	192	(66%)	87	(66%)	105	(66%)	1
	Age, mean (SD)	35.0	(8.4)	30.2	(7.5)	39.9	(6.0)	<0.0001	35.1	(7.4)	32.2	(7.1)	37.6	(6.6)	<0.0001
	AAO, mean (SD)	23.7	(9.2)	15.1	(1.6)	32.6	(3.7)	<0.0001	23.1	(5.8)	18.6	(2.4)	26.8	(5.0)	<0.0001
Co-affected siblings		n = 181		n = 90		n = 91									
	Male, n (%)	111	(61%)	57	(63%)	54	(59%)	0.64							
	Age, mean (SD)	34.8	(8.6)	30.6	(7.5)	38.9	(7.6)	<0.0001							
	AAO, mean (SD)	21.4	(4.8)	19.6	(3.4)	23.1	(5.3)	<0.0001							
Parents		n = 288	n = 156	n = 132											
	Male, n (%)	127	(44%)	73	(47%)	54	(41%)	0.34							
	Age, mean (SD)	63.1	(9.2)	59.3	(8.7)	67.7	(7.5)	<0.0001							
	Affected, n (%)	21	(7.3%)	13	(8.3%)	8	(6.1%)	0.50							
	AAO, mean (SD)	32.9	(11.0)	28.8	(8.7)	43.0	(14.8)	0.02							

AAO: age at onset.

### Genome-wide association study

**Figure 1:**
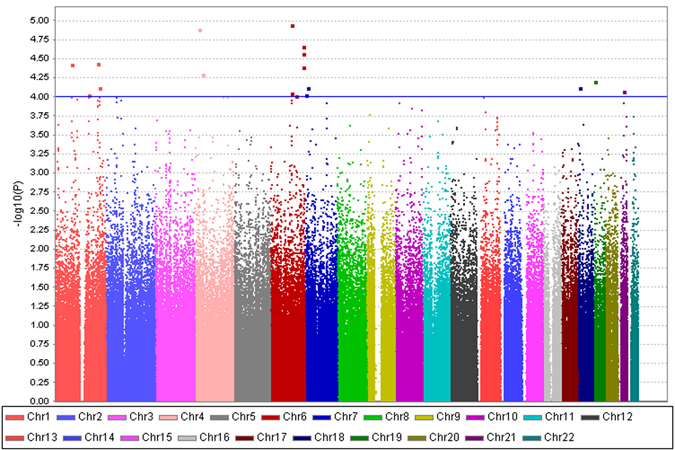
Manhattan plot of a genome-wide association study for the age at onset of schizophrenia performed in 94 probands from multiplex families with the earliest AAO and those with 91 the latest AAO. The x-axis is the chromosomal position, and the y-axis is the significance of the association represented by -log_10_(P-value) derived from multiple logistic regression analyses. The horizontal blue line shows the significance threshold at -log_10_(P-value) >4 for candidate SNPs.

The Manhattan plot of the initial GWAS analysis among schizophrenia probands from families with extreme contrast in AAO is displayed Fig. 1, with none reaching genome-wide significance. Nevertheless, 17 SNPs reached a suggestive threshold (P-values < 10^−4^). Table 2 displays the names, locations, minor allele frequencies (MAF), odds ratios (OR), and P-values of the association on 17 candidate SNPs, which are located on chromosome 1, 4, 6, 7, 18, 19, and 21. All the empirical P-values of the 17 SNPs remained less than 10^−4^ after 10^8^ times of permutation. Among these SNPs, 9 were mapped to the introns of 7 genes, whereas the remaining SNPs were in intergenic regions. Three intronic SNPs (rs3016537, rs7755434, and rs60117510) were mapped to the same intron in *PARK2*, spaced less than 300 base pairs apart with high linkage disequilibrium (LD); therefore, only one SNP (rs60117510) with the smallest P-value was included in the subsequent analyses.

**Table 2:** The SNPs with suggestive signal associated with age at onset of schizophrenia between different comparisons

Chr	SNP	Position	Gene	GWAS probands of extreme contrast in the AAO (n = 185)						Co-affected siblings of extreme contrast in the AAO (n = 181)				Trend of the AAO among 4 groups of probands^§^ (n = 475)	
				AL_m_	MAF_E_	MAF_L_	OR^a^	P-value	Empirical P-value	MAF_E_	MAF_L_	OR^a^	P-value	OR^b^	P-value
1	rs12118952	81,445,881	**ADGRL2**	A	0.25	0.46	0.38	3.8 × 10^−5^	1.6 × 10^−5^	0.30	0.40	0.66	0.06	0.66	8.9 × 10^−4^
1	rs6426994	166,361,616	*FMO7P*	G	0.30	0.49	0.39	9.6 × 10^−5^	5.5 × 10^−5^	—	—	—	—	—	—
1	rs609832	211,552,050	*RD3/SLC30A1*	G	0.18	0.04	6.32	3.6 × 10^−5^	5.3 × 10^−6^	0.14	0.07	2.44	0.02	1.95	8.9 × 10^−4^
1	rs2378013	218,778,121	*TGFB2*	A	0.41	0.20	2.68	7.5 × 10^−5^	3.6 × 10^−5^	0.33	0.26	1.44	0.13	1.62	5.9 × 10^−4^
4	rs923673	20,789,574	**KCNIP4**	C	0.30	0.12	4.08	1.3 × 10^−5^	3.2 × 10^−6^	0.25	0.17	1.69	0.06	1.69	3.4 × 10^−4^
4	rs11930588	40,179,089	**N4BP2**	T	0.49	0.30	3.02	5.1 × 10^−5^	1.6 × 10^−5^	0.43	0.36	1.41	0.14	1.46	1.7 × 10^−4^
6	rs2506754	103,548,429	*R3HDM2P2*	A	0.30	0.12	3.94	1.2 × 10^−5^	1.9 × 10^−6^	0.22	0.18	1.37	0.25	1.88	2.6 × 10^−3^
6	rs12210422	103,595,851	*R3HDM2P2*	G	0.35	0.15	3.01	8.9 × 10^−5^	3.8 × 10^−5^	0.30	0.25	1.31	0.26	1.64	5.5 × 10^−6^
6	rs6900852	125,901,926	**NCOA7**	G	0.37	0.43	0.39	9.8 × 10^−5^	5.2 × 10^−5^	0.42	0.49	0.73	0.14	0.62	1.0 × 10^−4^
6	rs3016537	161,816,611	**PARK2**	C	0.13	0.30	0.27	4.1 × 10^−5^	1.6 × 10^−5^	—	—	—	—	—	—
6	rs7755434	161,816,624	**PARK2**	A	0.12	0.29	0.26	2.7 × 10^−5^	9.6 × 10^−6^	—	—	—	—	—	—
6	rs60117510	161,816,881	**PARK2**	C	0.12	0.29	0.25	2.2 × 10^−5^	9.1 × 10^−6^	0.12	0.26	0.40	0.001	0.64	2.5 × 10^−3^
7	rs6964070	4,463,521	*SDK1/FOXK1*	G	0.53	0.35	2.63	9.3 × 10^−5^	4.5 × 10^−5^	0.49	0.36	1.73	0.01	1.51	1.1 × 10^−3^
7	rs1589988	16,227,085	**ISPD**	A	0.18	0.34	0.34	7.6 × 10^−5^	2.9 × 10^−5^	0.20	0.31	0.55	0.02	0.62	4.0 × 10^−4^
18	rs571002	13,090,667	**CEP192**	T	0.22	0.42	0.33	7.7 × 10^−5^	3.8 × 10^−5^	0.29	0.36	0.73	0.17	0.61	1.5 × 10^−4^
19	rs371164	6,489,802	*DENND1C/TUBB4A*	G	0.53	0.40	2.52	6.3 × 10^−5^	2.9 × 10^−5^	0.49	0.34	1.98	0.004	1.53	4.2 × 10^−4^
21	rs2830964	27,474,617	*RPL10P1/NCSTNP1*	C	0.22	0.39	0.34	8.4 × 10^−5^	3.8 × 10^−5^	0.27	0.32	0.72	0.19	0.65	8.0 × 10^−4^

Chr: chromosome; **bold**: SNP within the gene; *italics*: SNP near the gene; AL_m_: minor allele; AAO: age at onset; MAF_E_/MAF_L_: minor allele frequency in patients from families with earliest/latest AAO; OR^a^: odds ratio from association test using logistic regression; OR^b^: odds ratio from trend test using ordinal logistic regression. Empirical P presented the P-values obtained after up to 10^8^ times of permutation of the multiple logistic model. ^§^The trend effect of each SNP was tested using ordinal logistic regression among 4 groups of probands from families with earliest, earlier, later, and latest AAO of schizophrenia.

### Candidate SNPs in additional subsamples

These 15 SNPs were then genotyped in two additional samples: 1) the 181 co-affected siblings of the initial probands with extreme contrast in AAO and 2) the 290 unrelated probands from the remaining families with less contrast in average AAO (Table 2), with rs6426994 being excluded from analysis due to its low call rate. Among the co-affected sibling subsample from the families with extreme contrast in AAO, 5 SNPs exhibited significant associations (P-values < 0.05). Only the SNP on *PARK2* (rs60117510 with P < 0.001) remained significant after adjustment for multiple comparisons (P-value < 0.0036 = 0.05/14).

Regarding the 290 unrelated probands from the remaining families with less contrast in the AAO, none of the 14 SNPs reached statistical significance. Nevertheless, when the patients from the initial 185 probands with extreme contrast in the AAO and the 290 probands from the remaining families with less contrast in the AAO were examined as unrelated patients from four family subgroups (earliest, earlier, later, and latest AAO), all the 14 SNP genotypes showed a significant trend for the AAO (Table 2). The results of statistical models with adjustment for gender among the co-affected siblings and the unrelated gradient AAO groups remain similar (Supplementary Table S2).

### Multi-SNP genetic risk scores

**Figure 2:**
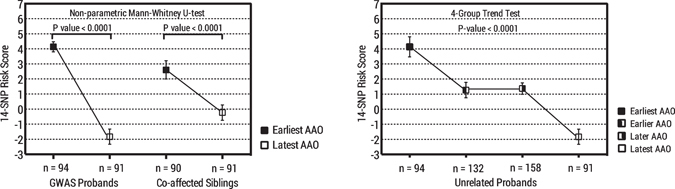
Multi-SNP genetic risk scores composed of the 14 SNPs associated with age at onset of schizophrenia. The left panel shows the difference of the 14-SNP genetic risk scores among both probands (94 with earliest and 91 with latest AAO) and co-affected siblings (90 with earliest and 91 with latest AAO) between two family groups, with a P-value < 0.0001 using the non-parametric Mann-Whitney U-test. The right panel shows a significant trend of the 14-SNP genetic risk scores among the probands from 4 family groups with a gradient AAO (earliest, earlier, later, and latest AAO), with a P-value < 0.0001 using the trend test using linear regression.

To obtain a multi-SNP GRS composed of the 14 candidate SNPs, the 185 probands with GWAS data were used as the learning set, in which the logOR were used as the weighting system. The probands with earliest AAO had greater 14-SNP GRS than those with latest AAO (left panel in Fig. 2). Then, the weights of the 14 SNPs were carried over to calculate the corresponding GRS among the 181 co-affected siblings of the GWAS probands, and the comparisons of the GRS between the two types of families remained significant, although the magnitude of contrast decreased (left panel in Fig. 2). Furthermore, the 14-SNP GRS for the 290 unrelated probands from the remaining families with less contrast in the AAO were calculated and used in the analysis of the unrelated patients from four family subgroups (earliest, earlier, later, and latest AAO), a significant trend of the GRS was observed (right panel in Fig. 2). All comparisons of the 14-SNP GRS maintained the statistical significance (P < 0.0001) with adjustment for gender (data not shown).

### AAO-associated SNPs and susceptibility

The relationship between the 14 AAO-associated SNPs and susceptibility to schizophrenia was analysed using two approaches (Table S1). First, the association between the 14 candidate SNPs and affliction with schizophrenia was examined using an additive model of the FBAT within the 185 multiplex families. The results showed that only 1 SNP (rs571002) had a weak association signal (P = 0.049). Second, by incorporating 925 age- and gender-matched community controls with the initial 185 schizophrenia probands, the conventional case-control association tests indicated that none of the 14 SNPs had different allele frequencies between the cases and the controls.

### Network analysis

Out of the 14 candidate SNPs identified associated with an early AAO, 7 were located within introns. These 7 SNPs were then subjected to the network analysis. The results revealed 5 significant gene networks with p-score >2, including a larger network centering on *PARK2* and 4 small networks consisting of *N4BP2*, and *KCNIP4*, *ADGRL2*, and *NCOA7*, respectively (Supplementary Figure S1). These 5 networks could be merged into a complicated scheme that may be involved in known diseases or biological functions, such as nervous system development, molecular transport, hereditary disorder, or cell-to-cell signaling and interaction. Detailed information on these networks is shown in Supplementary Table S3.

## Discussion

In this study, we identified 14 genetic loci represented by SNPs that were associated with an earlier AAO in a series of patients affected by schizophrenia from multiplex families with Han Chinese ancestry in Taiwan. Initially, 14 SNPs with the most significant association signals (P-value < 10^−4^) were obtained in a GWAS analysis for AAO among schizophrenia probands from two subgroups of families with extreme differences in the AAO (earliest AAO versus latest AAO). The association of the 14 SNPs were further evaluated in the co-affected siblings of the GWAS probands as well as in the probands from the families with less contrast in the AAO. When unrelated probands from four family subgroups (earliest, earlier, later, and latest AAO) were examined, all the 14 SNPs showed a significant trend for the AAO. A multi-SNP GRS consisting of the 14 candidate SNPs was able to distinguish probands with the earliest AAO from those with the latest AAO. Then, the discriminative validity of the GRS was further confirmed in the co-affected siblings of the GWAS probands as well as the unrelated probands from the four family subgroups characterized by an average AAO.

Some issues on our study design should be noted. First, this study primarily aimed to identify modifier loci associated with an earlier AAO of schizophrenia using a case-only design rather than susceptibility loci of schizophrenia using a case-control design. Second, we selected patients from families with contrast in AAO to conduct a dichotic comparison for association analyses instead of using patient onset age as a continuous outcome. Nevertheless, the linear trends detected for individual SNPs among the four ordinary AAO subgroups (earliest, earlier, later, and latest AAO) did indicate certain dosage effects. Third, limiting our patients to a single ethnicity may help decrease potential confounding effects from population admixture. Finally, adopting the PRS typically constructed from thousands of SNPs, we estimated a multi-SNP GRS composed of the 14 SNPs and confirmed its combined effect on the modification of the AAO across our family samples. The converging results from multiple lines of evidence provide robustness to our findings.

An important feature of this study is that we used patients from multiplex families with co-affected sib-pairs to search for modifier genetic loci associated with an early AAO in schizophrenia. Both an earlier AAO and a positive family history imply greater genetic contributions in these patients with schizophrenia^14, 15^. Given the same number of individuals that could be genotyped using GWAS chip under the constraints of resources, comparing two groups of multiplex schizophrenia patients with extreme contrast in AAO might have better power in detecting the associated genetic variants. The remaining co-affected sibling in each family become a genetically similar group for confirming the associations derived from the initial earliest vs. the latest onset probands. Using patients with relatively homogeneity in genetic loadings might also account for our success in confirming the initial hits from the GWAS results, which is typically more difficult in regular case-control studies due to heterogeneous background^6, 7^. In addition, we chose a threshold less stringent than the genome-wide significance level to select suggestive loci for further investigation, which has been adopted in many post-GWAS studies of schizophrenia^15, 32^. Nevertheless, the associations of these suggestive loci with AAO among the other co-affected siblings as well as multiplex patients from other families with less contrast in AAO lend consistent support to the initial selection.

Further, we applied a series of samples to confirm the association of 14 candidate loci with AAO in schizophrenia. The 2 groups of unrelated probands with less contrast of AAO was pooled into the initial 185 probands to conduct a gradient characteristic, considering the use of patients from multiplex families to reduce heterogeneity. Although not all individual SNPs conformed to the prediction in the samples consisting of patients with less contrast in the AAO, all of them exhibited a significant linear trend when pooling all 4 groups of unrelated probands together. With the gradient AAO among the 4 family groups, we substantiated that the AAO-modifying loci found in a dichotic design had implied a trend effect across the whole multiplex family sample. Nevertheless, alternative statistic models with adjustment for gender did not show evident changes in the significance of the association in single SNP and multi-SNP GRS analyses.

Among the 14 genetic loci we identified as AAO-associated in schizophrenia, 7 are intronic and another 7 are intergenic. The 7 SNPs or their surrounding regions within genes have been reported to be related to neurological disorders, such as schizophrenia^22, 33–37^ and Parkinson’s disease^38–40^ in previous GWLS and GWAS. The network analysis of the 7 annotated genes revealed 5 networks, each with *PARK2*, *N4BP2*, *KCNIP4*, *ADGRL2*, or *NCOA7* as its focus gene, which may have joint influence on the initiation of schizophrenia.

Though preliminary in nature, there have been clues about possible involvement of these genes in neurological diseases. First, *ADGRL2* at chromosome 1p31.1 had a nonparametric linkage Z score of 2.08 for marker D1S551 related to schizophrenia in the GWLS conducted in the original TSLS families^22^, indicating a consistent signal in both linkage and association studies. Intriguingly, the same linkage signal on 1p31.1 was also reported in a GWLS for schizophrenia in an Indian sample of 124 affected sib-pair families^33^. Second, a few SNPs on *KCNIP4* were suggestively associated with schizophrenia^34^, suicidal ideation in major depression^41^ and childhood ADHD^42, 43^ in GWAS, implying a function underlying these neuropsychiatric illnesses. Third, *N4BP2* at chromosome 4p14 was reported in linkage studies of schizophrenia^35^ and Parkinson’s disease^39^. Fourth, *NCOA7* was associated with Parkinson’s disease in GWAS^40^, and its expression was upregulated in the brain than in other human tissues^44^. Last but not least, mutations in *PARK2* at 6q25.2-q27 have been intensively studied in Parkinson’s disease, especially those with early onset^45^. There is increasing evidence pointing to a potential overlapping pathogenesis between Parkinson’s disease and schizophrenia since they had opposite dopaminergic dysfunctions, similar psychotic symptoms^46^, and shared some gene networks^47^.

Furthermore, 2 of the intergenic loci (SNP rs609832 and rs2378013) were predicted to have transcriptional regulation function, although the targets remained unclear. Unlike non-synonymous variants in exons, intronic and intergenic loci may serve as markers reflecting nearby genetic variants with functional meaning in schizophrenia^48^. Taken together, these genetic loci may have modifier functions for an earlier AAO in schizophrenia, although biological evidence is needed for further confirmation. The results of our GWAS for AAO in schizophrenia provide potential genetic markers and functional targets for future investigation.

While many GWAS for schizophrenia have been published, only two GWAS focusing on modifier loci responsible for AAO of schizophrenia have been conducted^14, 15^. Using schizophrenia patients of European ancestry, these two studies did not separate patients into those with and without family history when they analysed GWAS for the AAO. Each study identified multiple genetic loci that were associated with a quantitative AAO with a nominal significance level of P-value < 10^−6^ but could not validate them in the replication samples. Nevertheless, the loci identified by these two studies and our study did not overlap. One explanation for the inconsistent results could be the ethnic heterogeneity among studies^49^. Another explanation could be the difference in the scale of AAO, i.e., our use of subgroups with extreme contrast in AAO versus the use of continuous AAO in the other two studies.

It is noteworthy that none of the 14 AAO-modifying genetic loci found in this study were reported in the 108 genetic loci for schizophrenia susceptibility reported by an international collaborative effort^7^. This highlights a growing recognition that modifier genes may involve mechanisms in the pathogenesis of schizophrenia that are different from those of susceptibility genes^13^. The discovery of modifier genetic loci may also contribute to the phenomenon of missing heritability in the GWA results of schizophrenia^50^.

Some limitations should be noted in this study. First, the number of probands was small in the initial GWAS for the AAO of schizophrenia. Nevertheless, using multiplex patients of single ethnic group may help increase the homogeneity of our family sample. Second, an independent sample for replication would be required for further confirmation of our results. Of note, collecting multiplex families of sib-pairs co-affected with schizophrenia would be a substantial endeavour. Third, our network analysis was limited by both the restricted annotation of SNPs located within genes and the inability to incorporate the intergenic regions in the analysis. Finally, the functions of the AAO-modifying loci warrant further investigation.

In conclusion, this study identified 14 modifier loci that are associated with the AAO of schizophrenia, and a multi-SNP GRS composed of these 14 loci can distinguish patients with an early AAO from those with a late AAO. The use of co-affected siblings and unrelated patients with a gradient AAO for confirmation of the initial GWAS provided a novel strategy to overcome a drawback of the small sample. Future investigation of these AAO-modifying genetic loci may help further illuminate the pathogenesis of schizophrenia.

## Electronic supplementary material

Supporting information
