# Supplementary material for: Genetic loci associated with an earlier age at onset in multiplex schizophrenia
Source: Sci Rep. 2017 Jul 25;7:6486. doi: 10.1038/s41598-017-06795-8 (PMC5527118; doi:10.1038/s41598-017-06795-8)
Supplement: Supplementary file 1 — Supporting information [file 41598_2017_6795_MOESM1_ESM.doc]

**Supporting information**

**Supplementary tables and figures**

**Title:**

**Genetic loci associated with an earlier age at onset in multiplex schizophrenia**

**Authors:**

A. L. Woolston^1^, P. Hsiao^2^, P. Kuo^1,2,3^, S. Wang^1,4^, Y. Lien^5^, C. Liu^6,7^, H. Hwu^1,6^, T. Lu^1,8^, E. Chuang, L. Chang, C. Chen, J. Wu, M. T. Tsuang, and W. J. Chen

**Supplementary Table S1**. Family-based and community-based association tests on susceptibility in the 14 SNPs associated with the age at onset schizophrenia.

|  |  |  |  |  |  | **FBAT** | | | |  | **Case-Control** | | | |
| --- | --- | --- | --- | --- | --- | --- | --- | --- | --- | --- | --- | --- | --- | --- |
| **Chr** | **SNP** | **Position** | **Gene** | **AL_m_** |  | **MAF_F_** | **Fam#** | **χ2** | **P-value^a^** |  | **MAF_SZ_** | **MAF_NC_** | **OR** | **P-value^b^** |
| 1 | rs12118952 | 81,445,881 | **ADGRL2** | A |  | 0.36 | 74 | 0.01 | 0.94 |  | 0.36 | 0.33 | 1.12 | 0.35 |
| 1 | rs609832 | 211,552,050 | *RD3/SLC30A1* | G |  | 0.11 | 38 | 0.01 | 0.91 |  | 0.11 | 0.11 | 0.99 | 0.94 |
| 1 | rs2378013 | 218,778,121 | *TGFB2* | A |  | 0.33 | 72 | 2.17 | 0.14 |  | 0.31 | 0.29 | 1.05 | 0.72 |
| 4 | rs923673 | 20,789,574 | **KCNIP4** | C |  | 0.22 | 54 | 0.12 | 0.73 |  | 0.21 | 0.20 | 1.04 | 0.78 |
| 4 | rs11930588 | 40,179,089 | **N4BP2** | T |  | 0.41 | 81 | 0.39 | 0.53 |  | 0.40 | 0.42 | 0.93 | 0.52 |
| 6 | rs2506754 | 103,548,429 | *R3HDM2P2* | A |  | 0.21 | 64 | 1.32 | 0.25 |  | 0.21 | 0.21 | 0.93 | 0.64 |
| 6 | rs12210422 | 103,595,851 | *R3HDM2P2* | G |  | 08 | 65 | 0.03 | 0.87 |  | 0.25 | 0.29 | 0.80 | 0.09 |
| 6 | rs6900852 | 125,901,926 | **NCOA7** | G |  | 0.44 | 83 | 1.21 | 0.27 |  | 0.47 | 0.46 | 1.03 | 0.78 |
| 6 | rs60117510 | 161,816,880 | **PARK2** | C |  | 0.18 | 50 | 0.08 | 0.78 |  | 0.20 | 0.20 | 1.08 | 0.59 |
| 7 | rs6964070 | 4,463,521 | *SDK1/FOXK1* | G |  | 0.43 | 73 | 0.42 | 0.52 |  | 0.44 | 0.41 | 1.14 | 0.26 |
| 7 | rs1589988 | 16,227,085 | **ISPD** | A |  | 0.25 | 62 | 0.80 | 0.37 |  | 0.26 | 0.29 | 0.81 | 0.11 |
| 18 | rs571002 | 13,090,667 | **CEP192** | T |  | 0.34 | 76 | 3.88 | 0.049* |  | 0.32 | 0.32 | 1.01 | 0.93 |
| 19 | rs371164 | 6,489,802 | *DENND1C/TUBB4A* | G |  | 0.43 | 77 | 0.12 | 0.73 |  | 0.42 | 0.41 | 1.03 | 0.83 |
| 21 | rs2830964 | 27,474,617 | *RPL10P1/NCSTNP1* | C |  | 0.18 | 65 | 0.71 | 0.40 |  | 0.31 | 0.30 | 1.05 | 0.68 |

Chr: chromosome; **bold**: SNP within the gene; *italics*: SNP near the gene; AL_m_: minor allele; FBAT: family-based association test among 185 multoplex families of schizophrenia; MAF_F_: minora allele frequency of the family sample; Fam#: informative family numbers; χ2: chi-squared statistic; P-value^a^: P-value of the FBAT analyses; Case-Control: the case-control association tests between 185 schizophrenia patients and 925 age- and sex-matched normal controls; MAF_SZ_: minora allele frequency among schizophrenia patients; MAF_CN_: minora allele frequency among normal controls; OR: odds ratio; P-value^b^: P-value of the multiple logistic regression with adjustment for age and sex.

**Supplementary Table S2. The 14 SNPs with associations on age at onset of schizophrenia with adjustment for gender**

|  |  |  |  | **Co-affected siblings of extreme**  **contrast in the AAO**  **(n=181)** | |  | **Trend of the AAO among**  **4 groups of probands^§^**  **(n=475)** | |
| --- | --- | --- | --- | --- | --- | --- | --- | --- |
| **Chr** | **SNP** | **Position** |  | **OR^a^** | **P-value** |  | **OR^b^** | **P-value** |
| 1 | rs12118952 | 81,445,881 |  | 0.39 | 0.06 |  | 0.70 | 5.6E-03 |
| 1 | rs609832 | 211,552,050 |  | 9.51 | 0.047 |  | 1.93 | 9.5E-04 |
| 1 | rs2378013 | 218,778,121 |  | 1.44 | 0.49 |  | 1.71 | 8.3E-05 |
| 4 | rs923673 | 20,789,574 |  | 1.52 | 0.50 |  | 1.68 | 3.2E-04 |
| 4 | rs11930588 | 40,179,089 |  | 2.62 | 0.12 |  | 1.42 | 4.7E-03 |
| 6 | rs2506754 | 103,548,429 |  | 1.00 | 1.00 |  | 1.66 | 3.2E-04 |
| 6 | rs12210422 | 103,595,851 |  | 0.52 | 0.25 |  | 1.65 | 1.2E-04 |
| 6 | rs6900852 | 125,901,926 |  | 0.88 | 0.77 |  | 0.67 | 1.1E-03 |
| 6 | rs60117510 | 161,816,880 |  | 0.22 | 0.035 |  | 0.70 | 1.7E-02 |
| 7 | rs6964070 | 4,463,521 |  | 1.95 | 0.21 |  | 1.58 | 3.8E-04 |
| 7 | rs1589988 | 16,227,085 |  | 1.20 | 0.73 |  | 0.61 | 2.8E-04 |
| 18 | rs571002 | 13,090,667 |  | 1.03 | 0.95 |  | 0.70 | 6.3E-03 |
| 19 | rs371164 | 6,489,802 |  | 1.69 | 0.28 |  | 1.58 | 1.6E-04 |
| 21 | rs2830964 | 27,474,617 |  | 0.62 | 0.41 |  | 0.66 | 1.5E-03 |

Chr: chromosome; **bold**: SNP within the gene; *italics*: SNP near the gene; AL_m_: minor allele; AAO: age at onset; OR^a^: odds ratio from association test using logistic regression; OR^b^: odds ratio from trend test using ordinal logistic regression. ^§^The trend effect of each SNP was tested using ordinal logistic regression among 4 groups of probands from families with earliest, earlier, later, and latest AAO of schizophrenia.

**Supplementary Table S3**. Detailed information on 5 gene networks related to age at onset of schizophrenia

| **Focus gene** | **Molecules in network** | **Top diseases and functions** | **p-score** |
| --- | --- | --- | --- |
| N4BP2 | KAT5, N4BP2 | Cancer, Cell Death and Survival, Cellular Development | 3 |
| KCNIP4 | KCNIP4, KCNIP4-IT1, PSEN2 | Nervous System Development and Function, Organ | 3 |
|  |  | Development, Cell Signaling |  |
| ADGRL2 | ADGRL2, HAMP, MGEA5 | Cellular Function and Maintenance, Molecular Transport, | 3 |
|  |  | Cardiovascular System Development and Function |  |
| NCOA7 | EZH2, NCOA7, PRDM5 | Developmental Disorder, Hereditary Disorder, Ophthalmic | 3 |
|  |  | Disease |  |
| PARK2 | Abl1/2, ACTG1, AMBRA1, CCT2, CDK5, CUL1, | Cell-To-Cell Signaling and Interaction, Drug Metabolism, | 2 |
|  | EDNRB, FBXW7, FUBP1, GPR37, HSPA9, HSPD1, | Small Molecule Biochemistry |  |
|  | MAOA, MAOB, MFN1, MFN2, Mitochondrial |  |  |
|  | complex 1, PARK2, RNF31, RNF41, SARM1, SEPT4, |  |  |
|  | SNCA, STUB1, TCP1, TOMM20, TOMM40, |  |  |
|  | TOMM70A, TUBA1B, TUBB, TUBB2A, UBE2G2, |  |  |
|  | UBE2J1, UBE2L3, VDAC1 |  |  |

p-score: -log_10_(P-value).

**Supplementary Figure S1. The flow Chart of the study design and the sample selection.** GWAS: genome-wide association study; SZ(P): probands affected by schizophrenia; SZ(S): co-affected siblings of the probands; FBAT: family-based association tests.

**
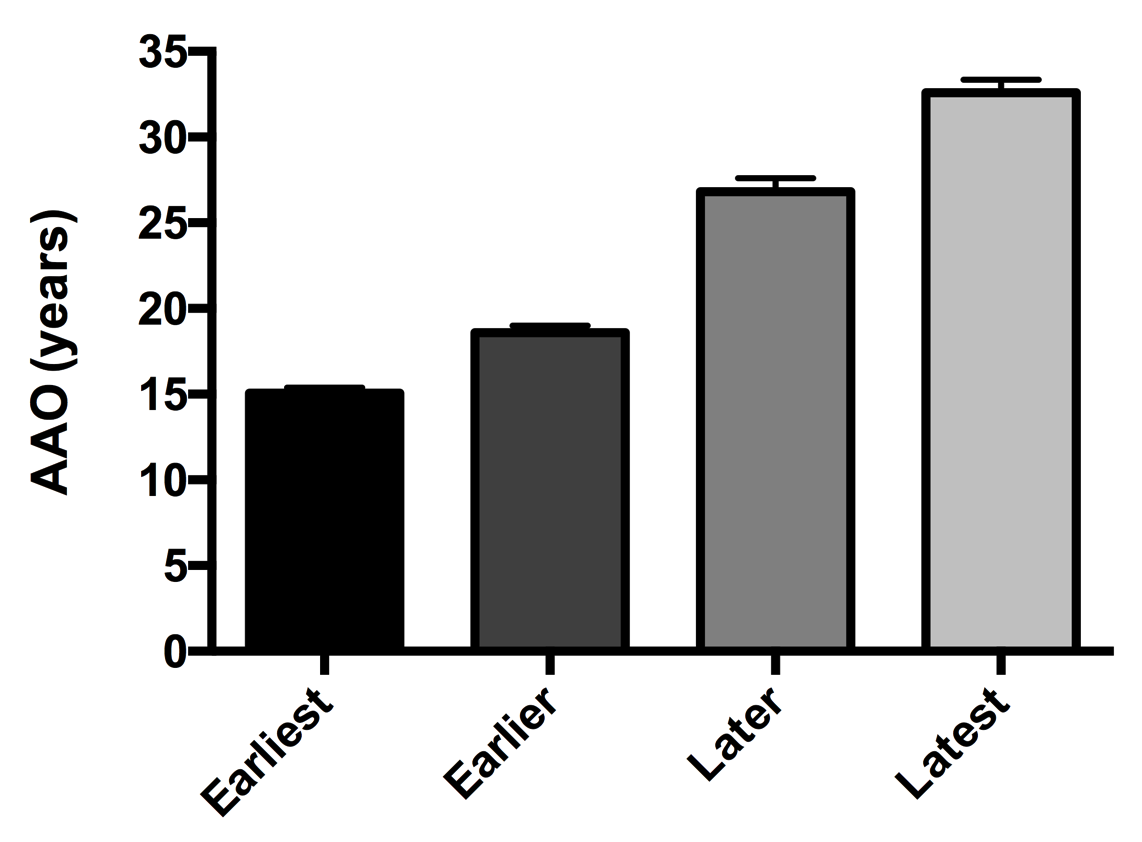
**

**Supplementary Figure S2. The age at onset of schizophrenia among probands from four family subgroups.** Four family subgroups of the earliest, earlier, later, and latest AAO are shown with a gradient distribution. AAO (years): the age at onset in years are described as mean with 95% confidence interval (CI).

**
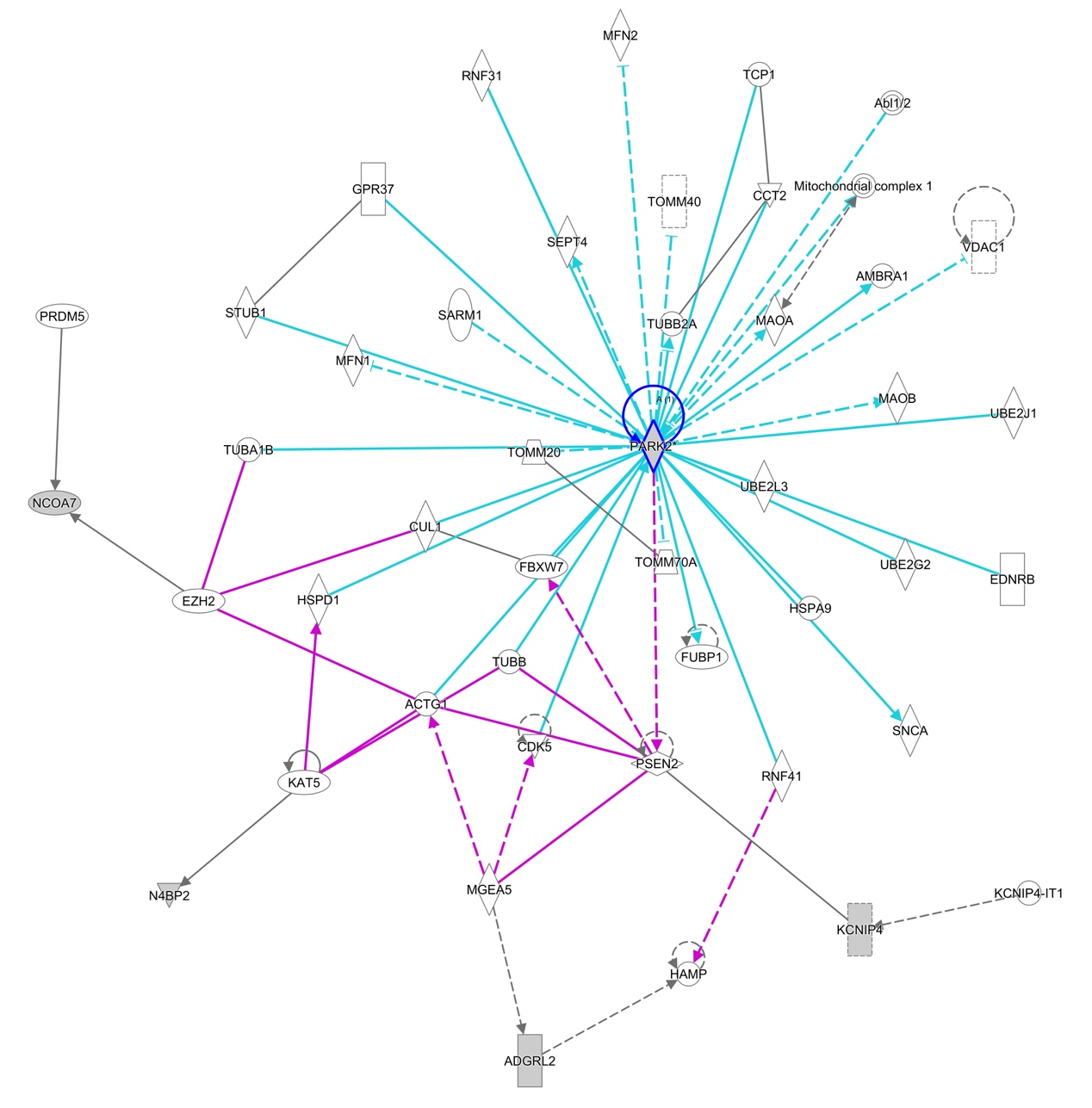
**

**Supplementary Figure S1. Five gene networks associated with the AAO of schizophrenia.** Gray shading: focus genes subjected to the network analysis; straight line: direct interaction; dotted line: indirect interaction; blue arrows indicate network of *PARK2*; pink arrows indicate key gene interaction between the networks.
